# Acute promyelocytic leukemia with the translocation t(15;17)(q22;q21) associated with t(1;2)(q42~43;q11.2~12): a case report

**DOI:** 10.1186/s13256-016-0982-8

**Published:** 2016-07-26

**Authors:** Abdulsamad Wafa, Faten Moassass, Thomas Liehr, Ayman Al-Ablog, Walid Al-Achkar

**Affiliations:** 1 Human Genetics Division, Department of Molecular Biology and Biotechnology, PO Box 6091, Damascus, Syria; 2 Institute of Human Genetics, Jena University Hospital, Kollegiengasse 10, D-07743 Jena, Germany

**Keywords:** Acute myeloid leukemia (AML), All-trans retinoic acid (ATRA), Acute promyelocytic leukemia (APL), t(1;2), Prognostic factors

## Abstract

**Background:**

Acute promyelocytic leukemia is characterized by a typical reciprocal translocation t(15;17)(q22;q21). Additional chromosomal abnormalities are reported in only 23–43 % of cases of acute promyelocytic leukemia.

**Case presentation:**

Here we report the case of a 46-year-old Syrian Alawis woman with acute promyelocytic leukemia with the typical t(15;17) translocation, but with a second clone presenting a t(1;2)(q42~43;q11.2~12) translocation as an additional abnormality. To the best of our knowledge, an association between these chromosomal abnormalities has not previously been described in the literature. Our patient started treatment with all-trans retinoic acid 10 days after diagnosis but died the same day of treatment initiation due to hemolysis, intracranial hemorrhage, thrombocytopenia, and disseminated intravascular coagulation.

**Conclusion:**

The here reported combination of aberrations in a case of acute promyelocytic leukemia seems to indicate an adverse prognosis, and possibly shows that all-trans retinoic acid treatment may be contraindicated in such cases.

## Background

Acute promyelocytic leukemia (APL) accounts for 5–10 % of acute myeloid leukemia (AML) and is a very distinct subtype (subtype M3) with regard to clinical, morphologic, and prognostic features. The median age of patients with APL is 30–40 years [1]. APL is characterized by the reciprocal translocation t(15;17)(q22;q21) in ~90 % of cases [1]. At the molecular level, as a result of the t(15;17) translocation, the gene for retinoic acid receptor alpha (*RARA*) on 17q21 fuses with a transcription factor gene (promyelocytic leukemia or *PML*) on 15q22, giving rise to a *PML/RARA* gene fusion product [2]. This *PML/RARA* fusion gene transcript is known to play a pivotal role in the pathogenesis of APL and the sensitivity to all-trans retinoic acid (ATRA) [3]. Approximately 70–80 % of patients with newly diagnosed APL carrying *PML/RARA* achieve long-term remission; however, some patients still have a poor outcome [3].

Balanced chromosomal rearrangements are detected in 25–30 % of adults with *de novo* AML [3, 4] and have attracted a great deal of attention because of specific translocations and inversions associated with the prognosis for these patients. Additional chromosomal aberrations (ACAs) associated with t(15;17) are reported in 23–43 % of APL cases [5–7]. The clinical impact of these ACAs has not yet been clearly elucidated.

Here we report the case of a patient exhibiting an immunophenotype consistent with APL, a t(15;17)(q22;q21) translocation, and a t(1;2)(q42~43;q11.2~12) translocation, with the clinical characteristics of hyperleukocytosis (HL), thrombocytopenia, and disseminated intravascular coagulation (DIC). Our patient did not benefit from ATRA treatment and died due to hemolysis, intracranial hemorrhage, thrombocytopenia, and DIC.

## Case presentation

A 46-year-old Syrian Alawis woman without a significant personal or familial medical history presented with a 1-month history of multiple sclerosis, fatigue, loss of weight, fever, and an elevated white blood cell (WBC) count. An initial evaluation revealed that she had anemia (8.5 g/dL), leukocytosis (total leukocyte count 134 × 10^9^/L), and thrombocytopenia (23 × 10^9^/L). She was pale and did not have lymphadenopathy.

Our patient was transferred to the hospital because she was unconscious and making noise during breathing. Novel hematological parameters included anemia (8.2 g/dL), thrombocytopenia (29 × 10^9^/L), leukocytosis (229 × 10^9^/L), a plasma concentration of fibrinogen of 37 mg/dL (normal value, 200–400 mg/dL), and a prothrombin time of 18 s (normal value, 10.0–13.0 s). She received several blood transfusions. Our patient stayed in the hospital for 1 week. On the same day of treatment initiation with ATRA (45 mg/m^2^ daily dose), our patient died, 10 days after her diagnosis. An autopsy revealed death was due to hemolysis, intracranial hemorrhage, thrombocytopenia, and DIC. Cytogenetic and immunophenotyping analyses were also carried out. Our patient was diagnosed with APL according to the World Health Organization (WHO) classification and was considered high risk based on her WBC. Her brother gave consent for a scientific evaluation of her case and the study was approved by the ethical committee of the Atomic Energy Commission, Damascus, Syria.

**Fig. 1:**
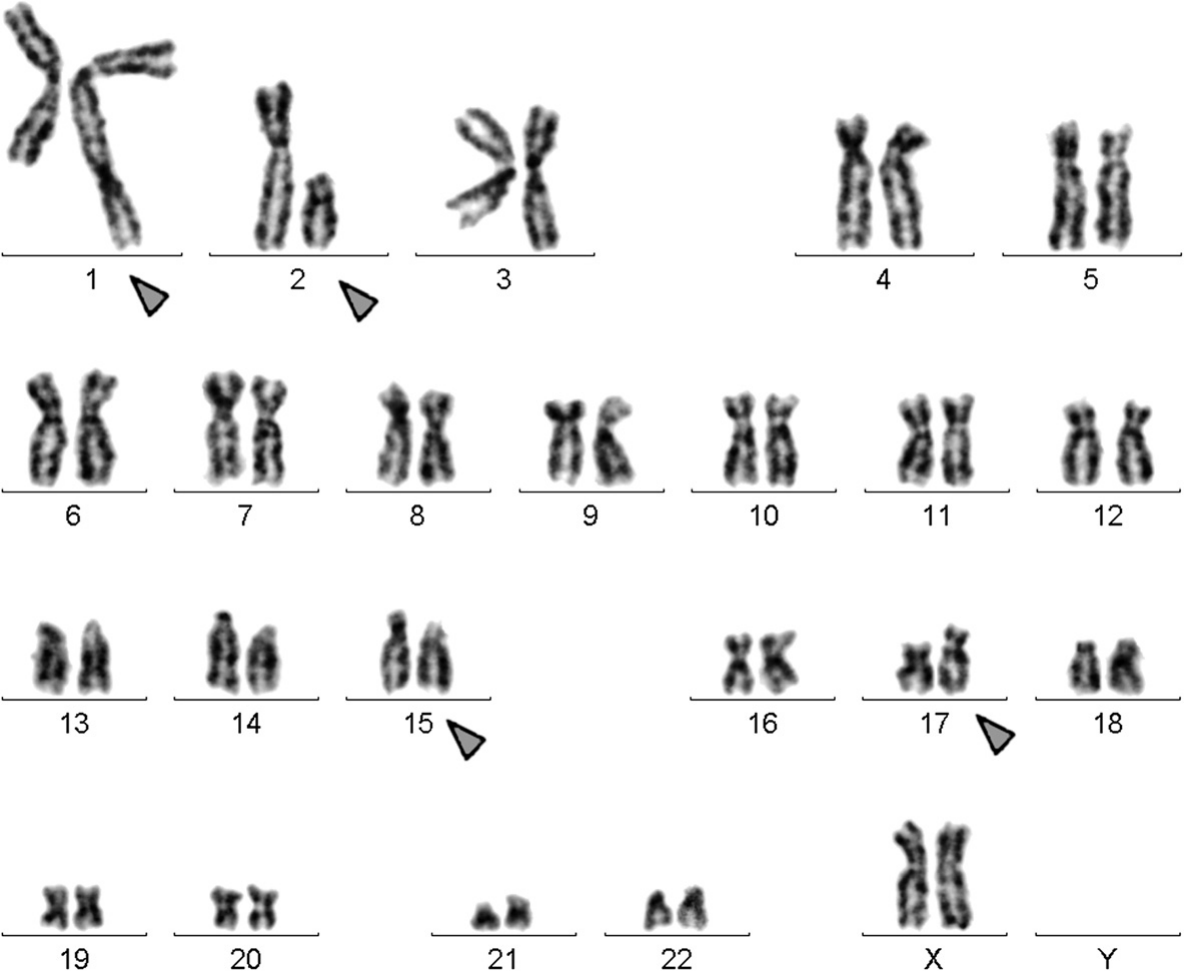
GTG-banding revealed the following karyotype: 46,XX,t(1;2)(q42~43;q11.2~12),t(15;17)(q22;q21). All derivative chromosomes are marked and highlighted by *arrow heads*

**Fig. 2:**
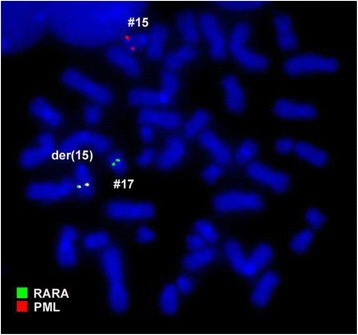
Fluorescence *in situ* hybridization using an LSI *PML*/*RARA* dual-color translocation probe for RARA (*green*) and PML (*red*) confirmed the presence of the *PML/RARA* fusion gene on der(15). *#* chromosome, *der* derivative chromosome

**Fig. 3:**
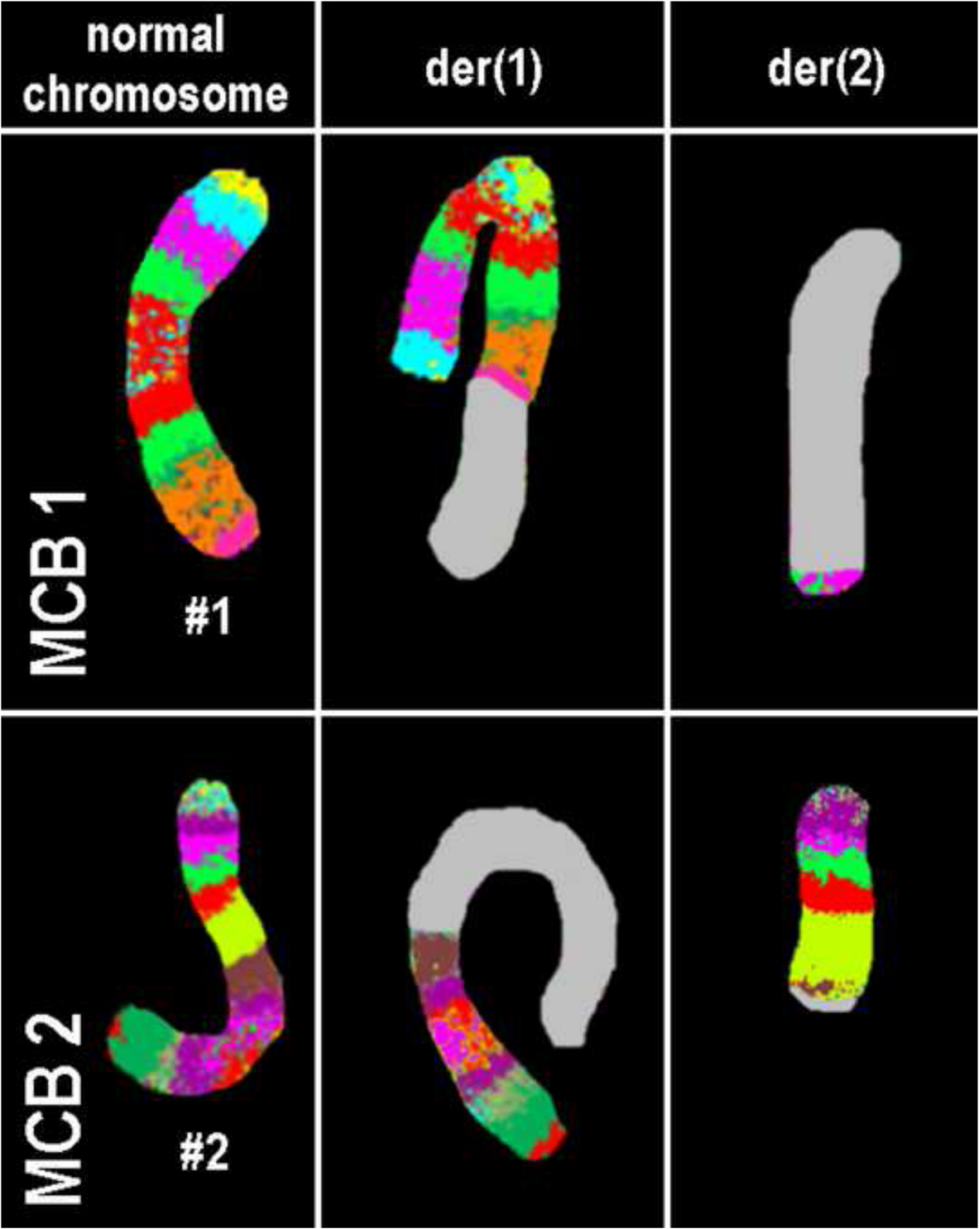
Array-proven multicolor banding (*aMCB*) was used to determine which chromosomes were involved in the present case. Each lane shows the results of aMCB analysis using probe sets for chromosomes 1 and 2. The normal chromosomes are shown in the first column and the derivatives of the two chromosomes in the subsequent ones. The unstained regions on the derivative chromosomes are shown in *gray*. # chromosome, *der* derivative chromosome

A chromosome analysis using GTG-banding was performed according to standard procedures [8] before treatment with ATRA and revealed a karyotype of 46,XX,t(15;17)[8]/46,XX,t(1;2),t(15;17)[11]/46,XX [1] (Fig. 1). Further studies were performed based on molecular cytogenetics (Figs. 2 and 3). Dual-color fluorescence *in situ* hybridization (D-FISH) using a specific probe for *PML* and *RARA* (Abbott Molecular/Vysis, Des Plaines, IL, USA) revealed the presence of the *PML*/*RARA* fusion gene on der(15) (Fig. 2). Chromosomes 1, 2, 15, and 17 were studied with Whole Chromosome Paint (WCP) probes (MetaSystems, Altlussheim, Germany) [8], which did not provide any information on the cryptic translocations (data not shown). Array-proven high-resolution multicolor banding (aMCB) [9] was performed using probes corresponding to chromosomes 1 and 2, which were identified by GTG-banding as being involved (Fig. 3). The following final karyotype was determined prior to chemotherapy treatment using a fluorescence microscope (AxioImager.Z1 mot, Carl Zeiss Ltd., Welwyn Garden City, UK) equipped with appropriate filter sets to discriminate between a maximum of five fluorochromes plus the counterstain DAPI (4',6- diamino-2-phenylindole). Image capture and processing were performed using an ISIS imaging system (MetaSystems, Altlussheim, Germany):

46,XX,t(15;17)(q22;q21)[8]/46,XX,t(1;2)(q42~43;q11.2~12),t(15;17)(q22;q21)[11]/46,XX[1].

Image capture and processing were performed using an ISIS imaging system (MetaSystems).

Immunophenotyping was performed using a general panel of fluorescent antibodies against the following antigens typical for different cell lineages and cell types: CD1a, CD2, CD3, CD4, CD5, CD8, CD10, CD11b, CD11c, CD13, CD14, CD15, CD16, CD19, CD20, CD22, CD23, CD32, CD33, CD34, CD38, CD41a, CD45, CD56, CD57, CD64, CD103, CD117, CD123, CD138, CD209, CD235a, and CD243. In addition, antibodies to kappa and lambda light chains, IgD, sIgM, and HLADr were tested. All antibodies were purchased from BD Biosciences, San Jose, CA, USA. Samples were analyzed on a BD FACSCalibur™ flow cytometer. Autofluorescence, viability, and isotype controls were included. Flow cytometric data acquisition and analysis were conducted by BD Cellquest™ Pro software. Flow cytometric analysis of a peripheral blood specimen from our patient characterized this case as APL according to WHO classifications. The abnormal cell population (97 % of tested cells) was positive for MPO^++^, CD45^+dim^, CD34^−^, HLADr^−^, CD33^+^, CD13^+^, CD16^−^, CD64^+^, CD15^+dim^, and CD14^−^.

## Discussion

According to the literature, APL is characterized by the t(15;17) translocation that generates the *PML/RARA* fusion gene and induces sensitivity to ATRA [3]. To date, 1402 APL cases with t(15;17) have been reported in the Mitelman Database [10]. Only three cases demonstrated involvement of a t(1;2) translocation in addition to t(15;17): the first case was a t(1;2)(p22;q31), the second a t(1;2)(q43;p21), and the third a t(1;2;3)(p36;q21;p21) [10]. To the best of our knowledge, this is the first report of a case of APL with t(15;17)(q22;q21) associated with t(1;2)(q42~43;q11.2~12).

In addition, chromosomal band 1q42 is reported in two cases, 1q43 in one, and 2q12 in one case in the Mitelman Database [10]. Chromosomal band 2q11.2 has not yet been reported in APL [10].

Additional chromosome aberrations to t(15;17) have been observed in 23–43 % of APL cases, but their prognostic significance remains controversial [5–7]. The majority of evidence supports the concept that patients with additional chromosomal abnormalities have the same favorable prognosis as patients with t(15;17) alone [5, 7]; however, a previous study has found that chromosomal abnormalities in addition to t(15;17) are associated with a poorer prognosis [6].

In contrast, another study showed that additional chromosomal abnormalities are associated with a slightly better prognosis (no effect on overall survival) [11]. Moreover, some reports found that ACAs had no effect on prognosis [7, 12]. However, some newly diagnosed patients and patients with relapsing disease with identical cytogenetic changes showed an adverse outcome [6, 13]. The most frequent secondary aberration to t(15;17) is trisomy 8 (+8). Other additional chromosome changes include del(9q); del(7q); abnormalities of chromosome 1, 3, and 6; trisomy 21; and isochromosome of the long arm of the derivative chromosome 17 originating from the translocation t(15;17) [ider(17)(q10)t(15;17) or ider(17q)] [5].

Zaccaria *et al*. [14] reported that a patient with APL associated with a *PML/RARA* fusion gene on chromosome 17 responded poorly to ATRA treatment. However, complete remission rates are usually 87–94 % using ATRA alone at a classical dosage of 45 mg/m^2^/day for 4–6 weeks [15].

Thus, it is not clear whether the novel cytogenetic findings in the present case relate to a slower than usual response to ATRA induction therapy. Furthermore, the presence of specific ACAs associated with translocation t(15;17) might be indicative of a poor outcome.

Clinically, APL has a high frequency of hemorrhage due to DIC, which contributes to the high mortality rates of this disease [16, 17]. However, DIC is a coagulopathy induced by the formation of small clots consuming coagulation proteins and platelets, resulting in disruption of normal coagulation and severe bleeding tendency [18]. Acute DIC is characterized by a decrease in platelet count and fibrinogen, an elevation of D-dimers, and prolongation of prothrombin time and activated partial thromboplastin time; it occurs in 30–40 % of HL-AML [19].

Five to twenty percent of patients with untreated AML present with HL, that is, WBC counts of >100,000 cells/mL [20]. HL may cause three main complications: (i) DIC, (ii) tumor lysis syndrome, and (iii) leukostasis. These may cause life-threatening complications in patients with AML [19]. Early mortality in this patient group is higher than in AML without HL and ranges from 6 % versus 1 % after 1 week and 13 % versus 7 % after 30 days [19]. The main causes of death are bleeding, thromboembolic events, and neurologic and pulmonary complications [21]. HL is a negative prognostic factor, as indicated by significantly shorter overall survival [22].

Approximately 44–50 % of patients with AML with a WBC count >100,000 cells/mL have a high probability of leukostasis. Organs most frequently affected are lung, brain, and kidneys [20]. As well as the tissue damage caused by stasis and leukocyte infiltration, hemorrhage and thromboembolic events are frequent and relevant complications of leukostasis [18].

## Conclusions

Here, we have described a case of APL characterized by HL, thrombocytopenia, and DIC associated with translocation t(15;17) and translocation t(1;2)(q42~43;q11.2~12). Because translocation t(15;17) is normally successfully treatable with ATRA even when ACAs are present, the adverse outcome in the present patient was surprising. Thus, the translocation t(1;2)(q42~43;q11.2~12) may be a new predictor for a more severe course of APL.

## Abbreviations

ACAs, additional chromosomal aberrations; aMCB, array-proven high-resolution multicolor banding; AML, acute myeloid leukemia; APL, acute promyelocytic leukemia; ATRA, all-trans retinoic acid; DAPI, (4′,6- diamino-2-phenylindole); D-FISH, dual-color fluorescence *in situ* hybridization; DIC, disseminated intravascular coagulation; HL, hyperleukocytosis; *PML,* promyelocytic leukemia gene; *RARA*, retinoic acid receptor alpha gene; WBC, white blood cell; WHO, World Health Organization
